# Geographic and economic influences on benralizumab prescribing for severe asthma in Japan

**DOI:** 10.1038/s41598-024-65407-4

**Published:** 2024-07-02

**Authors:** Nobuaki Kobayashi, Hiromi Matsumoto, Kohei Somekawa, Ayami Kaneko, Nobuhiko Fukuda, Suguru Muraoka, Yukiko Ohtsu, Momo Hirata, Ryo Nagasawa, Sousuke Kubo, Kota Murohashi, Hiroaki Fujii, Ayako Aoki, Keisuke Watanabe, Nobuyuki Horita, Yu Hara, Takeshi Kaneko

**Affiliations:** Department of Pulmonology, Yokohama City University Graduate School of Medicine, 3-9 Fukuura, Kanazawa-ku, Yokohama, Kanagawa 236-0004 Japan

**Keywords:** Severe asthma, Benralizumab, Access to care, Healthcare disparities, Socioeconomic factors, Asthma, Epidemiology

## Abstract

Benralizumab, a monoclonal antibody targeting IL-5 receptors, reduces exacerbations and oral corticosteroid requirements for severe, uncontrolled eosinophilic asthma. In Japan, geographic disparities in asthma outcomes suggest differential prescribing and access. This study aimed to quantify regional prescribing variations for benralizumab nationwide. Using Japan’s National Database (NDB) of insurance claims (2009–2019), benralizumab standardized claim ratios (SCRs) were calculated for 47 prefectures. Correlations between SCRs and other biologics’ SCRs, economic variables like average income, and physician densities were evaluated through univariate analysis and multivariate regressions. Income-related barriers to optimal prescribing were examined. Wide variation emerged in benralizumab SCRs, from 40.1 to 184.2 across prefectures. SCRs strongly correlated with omalizumab (r = 0.61, p < 0.00001) and mepolizumab (r = 0.43, p = 0.0024). Average monthly income also positively correlated with benralizumab SCRs (r = 0.45, p = 0.0016), whereas lifestyle factors were insignificant. Respiratory specialist density modestly correlated with SCRs (r = 0.29, p = 0.047). In multivariate regressions, average income remained the most robust predictor (B = 0.74, p = 0.022). Benralizumab SCRs strongly associate with income metrics more than healthcare infrastructure/population factors. Many regions show low SCRs, constituting apparent prescribing gaps. Access barriers for advanced asthma therapies remain inequitable among Japan’s income strata. Addressing affordability alongside specialist allocation can achieve better prescribing quality and asthma outcomes.

## Introduction

Severe asthma is characterized by chronic airway inflammation that remains inadequately controlled despite high-dose inhaled corticosteroids (ICS) and additional controller medications, with or without systemic corticosteroids^1^. In the literature, reported prevalence rates for severe asthma range widely from 1.8 to 38% of individuals with asthma, likely reflecting differences in study populations and case definitions^2^. The persistence of symptoms in such cases highlights the clinical challenge of managing this patient population and underscores the need for alternative treatment strategies.

Benralizumab (BRZ), a humanized monoclonal antibody, targets the interleukin-5 receptor alpha (IL-5Rα) on eosinophils, thereby reducing eosinophilic inflammation central to asthma pathogenesis. In Japan, BRZ was added as a standard treatment for severe asthma following Omalizumab, an IgE antibody that specifically targets immunoglobulin E to reduce allergic asthma symptoms, and Mepolizumab, an antibody against IL-5, which plays a critical role in the development and activation of eosinophils. Clinical trials show BRZ can decrease exacerbations and oral corticosteroid requirements, while improving lung function for patients with severe, uncontrolled eosinophilic asthma^3,4^. However, BRZ is not indicated or effective for all severe asthma patients^5^. Treatment guidelines recommend BRZ for those with blood eosinophil counts ≥ 300 cells/μL or ≥ 2 exacerbations requiring systemic corticosteroids in the prior year. Furthermore, the decision to prescribe BRZ may be influenced by a range of other factors, encompassing patient preferences, comorbidities, cost-efficiency, and availability^6–8^.

Therefore, we aimed to quantify regional differences in BRZ prescribing patterns for severe asthma patients in Japan using the National Database (NDB). Established in 2009, the NDB contains reimbursement claims from healthcare institutions across Japan. Analyzing this dataset, we investigated potential associations of BRZ prescription with patient demographics and health system variables.

## Results

### Regional difference in the number of asthma patients and standardized claim ratio of benralizumab in Japanese prefectures

In assessing the regional variation in asthma management in Japan, a detailed analysis of the number of asthma patients and the Standardized Claim Ratio (SCR) of BRZ was conducted, as illustrated in Table 1. The data reveals significant inter-prefectural variability in both the number of patients treated and the SCR for BRZ, Omalizumab, and Mepolizumab. In Japan, the healthcare system is underpinned by universal public health insurance, ensuring affordability and equal access to medical services for all citizens. Under this system, patients pay only a portion of medical expenses determined by their income level, while the remaining costs are covered through the public insurance scheme. Importantly, the fees for medical consultations, procedures, and treatments like BRZ are uniformly regulated nationwide. This obligates all hospitals and clinics to offer services at the established rates, regardless of institution or geographic location. Furthermore, BRZ can be prescribed by respiratory specialists as well as general practitioners for eligible patients. This universal coverage coupled with broad prescribing eligibility aims to facilitate access to advanced asthma therapies across Japan. However, the wide ranges observed in the SCRs suggest potential disparities in the actual utilization of BRZ between prefectures.

**Table 1:** Numbers of patients and SCR of drugs in each prefecture.

Prefecture	Population (millions, 2020)	Number of patients (thousand, 2020)	SCR (2020)		
			Omalizumab	Mepolizumab	Benralizumab
Hokkaido	5.25	3.5	101.1	80.2	124.3
Aomori	1.25	0.7	51.0	50.2	91.9
Iwate	1.23	0.8	88.0	91.9	93.4
Miyagi	2.31	1.3	79.7	77.8	98.1
Akita	0.97	0.4	125.1	81.2	63.2
Yamagata	1.08	0.6	50.1	50.9	51.5
Fukushima	1.85	1.1	73.8	70.3	65.9
Ibaraki	2.86	2.2	104.1	54.8	184.2
Tochigi	1.93	1.4	74.0	72.2	95.9
Gunma	1.94	2.4	72.6	101.8	96.8
Saitama	7.35	7.1	60.7	71.8	45.1
Chiba	6.26	2.7	71.7	80.3	77.4
Tokyo	13.92	10.1	197.4	264.3	179.2
Kanagawa	9.20	11.2	120.9	117.8	91.0
Niigata	2.22	1.3	84.2	55.4	98.9
Toyama	1.04	0.7	115.1	76.3	120.9
Ishikawa	1.14	0.5	60.5	117.7	94.1
Fukui	0.77	0.4	130.9	66.9	155.4
Yamanashi	0.81	0.3	81.6	50.4	83.5
Nagano	2.05	1.3	47.7	66.7	85.0
Gifu	1.99	1.3	76.6	71.9	72.2
Shizuoka	3.64	2.2	104.8	73.2	92.2
Aichi	7.55	5.1	108.7	79.2	93.3
Mie	1.78	1	79.8	65.6	80.1
Shiga	1.41	0.8	66.8	125.9	111.4
Kyoto	2.58	1.7	192.1	164.3	114.3
Osaka	8.81	4.4	95.5	104.2	97.2
Hyogo	5.47	3.8	88.8	98.9	82.1
Nara	1.33	0.7	86.3	60.9	93.2
Wakayama	0.93	0.5	70.5	68.6	69.8
Tottori	0.56	0.4	78.9	62.7	149.4
Shimane	0.67	0.6	94.1	42.4	96.2
Okayama	1.89	1.1	98.2	120.1	141.6
Hiroshima	2.80	3.4	96.5	95.8	119.7
Yamaguchi	1.36	0.9	75.4	74.0	102.0
Tokushima	0.73	0.5	42.3	44.4	43.3
Kagawa	0.96	0.6	143.8	57.6	122.2
Ehime	1.34	0.9	74.3	55.2	114.4
Kochi	0.70	0.5	41.3	77.4	40.1
Fukuoka	5.10	2.9	77.0	65.5	89.8
Saga	0.82	0.6	38.8	43.6	64.8
Nagasaki	1.33	1.8	84.8	52.7	57.6
Kumamoto	1.75	0.9	90.9	52.9	72.2
Oita	1.14	0.8	63.3	41.0	69.9
Miyazaki	1.07	0.9	83.7	93.8	57.7
Kagoshima	1.60	1.5	35.6	60.1	60.4
Okinawa	1.45	0.9	42.5	45.3	72.9

*SCR* standardized claim ratio, *BRZ* Benralizumab.

Ibaraki Prefecture emerged as the region with the highest SCR for BRZ at 184.2, suggesting a greater than average prescription rate when normalized against the national level, whereas Kochi Prefecture recorded the lowest SCR at 40.1. This demonstrates a remarkable difference, with the highest SCR being approximately 4.59 times greater than the lowest. Furthermore, Tokyo, with the largest patient population (10.1 thousand), showed a disproportionately high SCR for Omalizumab (197.4) and Mepolizumab (264.3), reflecting a preference or greater accessibility to these treatment options in the metropolitan area. Conversely, Kagoshima Prefecture, despite having a moderate patient population size (1.5 thousand), reported the lowest SCRs across all three medications.

**Figure 1:**
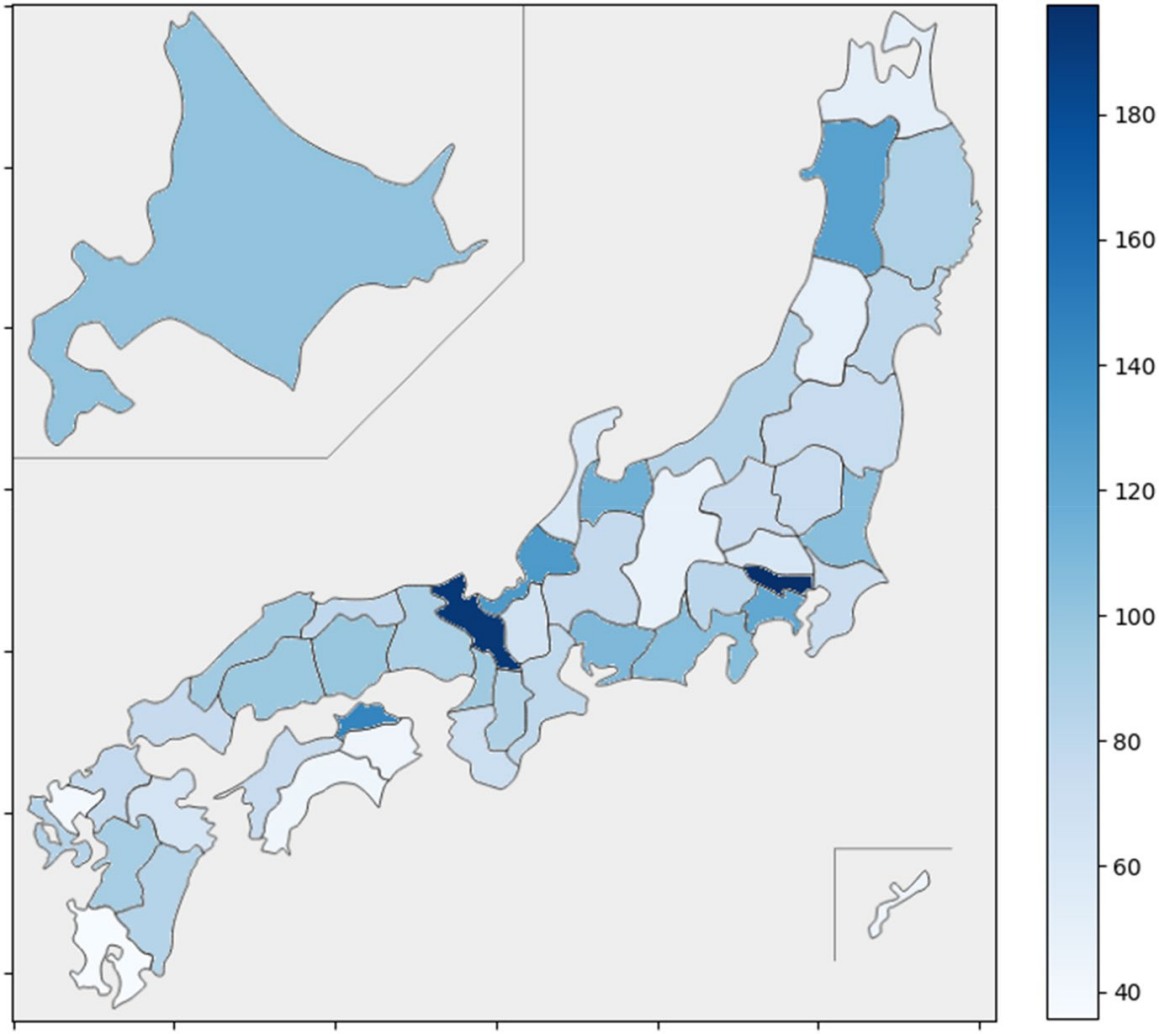
Intensity map showing SCR for each prefecture. The darker the color, the larger the SCR. A part of Hokkaido prefecture and Okinawa prefecture is different from the actual position. This figure was created by the authors using the ‘japanmap’ library (version 0.3.1) and ‘matplotlib’ library (version 3.9.1).

The intensity map (Fig. 1) presents a detailed visualization of the SCR for BRZ across the prefectures in Japan, highlighting significant inter-prefectural differences. Notably, the shades of gray depict the variance in each prefecture’s prescription rates relative to the national average, with darker tones indicating a higher SCR. Upon broader regional analysis, however, the data do not reveal a distinct pattern in the distribution of SCR between the larger northern and southern areas of Japan. Despite the notable disparities at the prefectural level, the absence of a clear gradient or trend when assessing larger geographical entities suggests that factors influencing BRZ prescription rates may not be related to these broad regions’ characteristics.

Such disparities in SCR of BRZ among different regions might reflect the accessibility, affordability, physician availability, preferences for biologics, or acceptance of this specific treatment and may warrant further investigation to ensure equitable care across all prefectures.

### Univariate analysis of factors related to SCR of benralizumab

**Figure 2:**
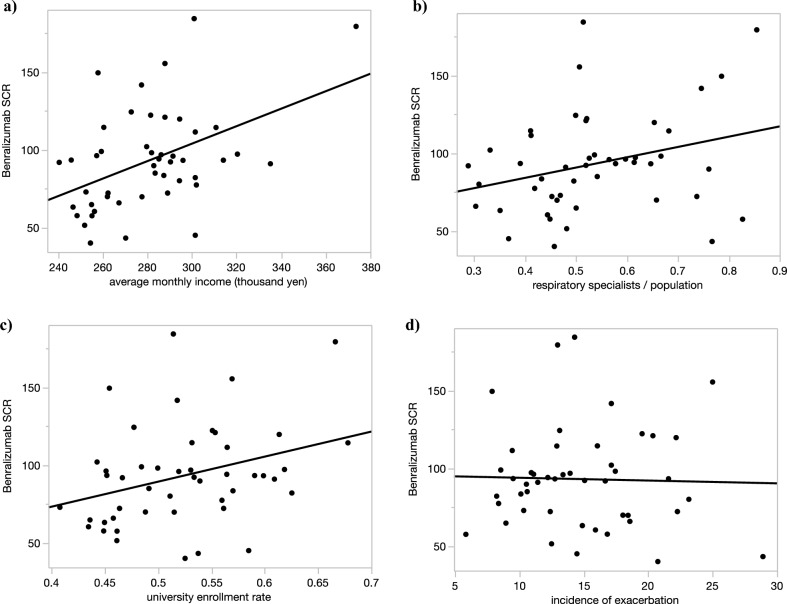
Scatter plot with the value of each explanatory variable on the x-axis and SCR on the y-axis. (**a**) The explanatory variable is the average monthly income. y = − 64, 5 − 0.56x, *R*^2^ = 0.20. (**b**) The explanatory variable is the number of respiratory specialists per population. y = 57.8 + 66.1x, *R*^2^ = 0.085. (**c**) The explanatory variable is the university enrollment rate. y = 9.0 + 160.7x, *R*^2^ = 0.10. (**d**) The explanatory variable is the incidence of exacerbation. y = 95.7 − 0.18x, *R*^2^ = 0.00078. *R*^2^ coefficient of determination, *SCR* standardized claim ratio.

The univariate analysis presented associations between BRZ SCRs and several demographic, economic, and healthcare factors across Japanese prefectures (Table 2). This analysis indicates a significant positive correlation with the SCR of Omalizumab (median: 79.8, range 35.6–197.4) and Mepolizumab (median: 71.8, range 41.0–264.3), with correlation coefficients of 0.61 (p < 0.00001) and 0.43 (p = 0.0024) respectively. The average monthly income (median: 281.9, range 240.5–303.6) also shows a positive correlation with a coefficient of 0.45 (p = 0.0016), which may reflect economic influences on the availability or the prescribing patterns of BRZ. Figure 2a indicates that average monthly income has a small negative impact on SCR, explaining 20% of its changes. Notably, the smoking rate among individuals aged 40 and above (median: 0.22, range 0.19–0.28), and the obesity rate (median: 0.29, range 0.26–0.40), are not significantly correlated with BRZ SCR. This could imply that lifestyle factors may not directly affect the prescription rates of this specific treatment within the studied regions.

**Table 2:** Correlation between SCR and each factor in 2020.

Factor	Median (range)	Correlation coefficient	p-value
SCR of omalizumab	79.8 (35.6–197.4)	0.61	< 0.00001
SCR of mepolizumab	71.8 (41.0–264.3)	0.43	0.0024
Average monthly income (thousand yen)	281.9 (240.5–303.6)	0.45	0.0016
Smoking rate (age 40 and above)	0.22 (0.19–0.28)	− 0.049	0.74
Obesity rate (age 40 and above, BMI ≥ 25)	0.29 (0.26–0.40)	− 0.27	0.069
Respiratory specialists (per population)	0.51 (0.29–0.85)	0.29	0.047
Allergy specialists (per population)	0.12 (0.03–0.33)	0.16	0.27
University enrollment rate	0.52 (0.41–0.68)	0.32	0.028
Proportion with steroid-requiring exacerbations	13.9 (5.9–28.9)	− 0.03	0.85

*BMI* body mass index, *SCR* standardized claim ratio, *r* Pearson’s correlation coefficient.

*p < 0.05.

The density of respiratory specialists per population (median: 0.51, range 0.29–0.85) presents a mild positive correlation (r = 0.29, p = 0.047), suggesting that the presence of healthcare professionals specializing in respiratory diseases may have a modest impact on BRZ prescription rates. The university enrollment (median: 0.52, range 0.41–0.68) rate also has a positive correlation (r = 0.32, p = 0.028), which might be indicative of the role of educational attainment in healthcare access or decision-making regarding advanced therapies. Scatter plots indicate associations between the SCR of BRZ and respiratory specialist density (Fig. 2b), or university enrollment rates (Fig. 2c). Figure 2b models the association between respiratory specialist density and BRZ SCR, with a regression slope of 66.1 and R^2^ of 0.085. Figure 2c examines the link with university enrollment rates, finding a positive correlation (slope = 160.7) that accounts for 10% of SCR variability (R^2^ = 0.10).

In Fig. 2d, a scatter plot with the incidence of exacerbations as the independent variable, presents a regression line characterized by y = 95.7 − 0.18x and an R^2^ of 0.00078. This strikingly low coefficient of determination underscores the absence of a meaningful relationship between the frequency of asthma exacerbations and the use of BRZ. This raises a significant concern regarding the adequacy of BRZ prescription for patients with severe asthma who experience frequent exacerbations—a patient group that would typically benefit from biologic treatments. The data suggest a potential underutilization of BRZ in these patients, indicating a gap in the management of severe asthma that warrants urgent attention.

### Multivariate analysis of factors related to SCR of benralizumab

To identify independent predictors of regional differences in BRZ standardized claim ratios (SCRs), we conducted a multivariate regression analysis including respiratory specialist density, average monthly income, university enrollment rates, and exacerbation rates requiring steroids (Table 3).

**Table 3:** Multiple regression analysis with SCR as the objective variable.

Explanatory variables	B (95% CI)	*p*	*β*	VIF	*R*^2^
Respiratory specialists (per population)	47.88 (− 16.31 to 112.08)	0.14	0.21	1.10	0.25
Average monthly income	0.74 (0.11 to 1.37)	0.022	0.59	3.48	
University enrollment rate	− 115.91 (− 371.40 to 139.57)	0.37	− 0.23	3.60	
Proportion with steroid-requiring exacerbations	0.10 (− 1.68 to 1.88)	0.91	0.016	1.07	

*95% CI* 95% confidence interval, *B* partial regression coefficient, *β* standardized partial regression coefficient, *VIF* variance inflation factor, *R*^*2*^ coefficient of determination, *SCR* standardized claim ratio.

*p < 0.05.

The number of respiratory specialists per population did not emerge as a significant independent predictor (B = 47.88, 95% CI [− 16.31 to 112.08], p = 0.14) after adjusting for the other factors. However, average monthly income remained significantly associated with SCRs (B = 0.74 [95% CI 0.11–1.37], p = 0.022) with the highest standardized regression coefficient (β = 0.59), indicating it had the greatest relative effect on BRZ prescription rates. Though income, specialists, and education had correlations in univariate tests, the multivariate results suggest income bears the most robust predictive relationship with BRZ usage patterns across regions.

University enrollment rates and exacerbation rates showed negligible regression coefficients in the multivariate model. Collectively, these results underline pronounced variability among prefectures explained predominantly by income-related factors, rather than healthcare infrastructure or population characteristics. Strategies addressing financial barriers may be warranted alongside specialist resource allocation to achieve more equitable BRZ prescribing practices.

## Discussion

The present study is to understand the regional disparities in the prescription of BRZ among patients with severe asthma in Japan. The observed regional differences in the prescription of BRZ have been identified in this study, with the range of SCR (40.1–184.2) demonstrating a substantial variance across different prefectures. A significant association was observed between the SCR of BRZ and the average monthly income in various prefectures.

Our findings emphasize the vital role of average monthly income in the prescription pattern of BRZ. The significant correlation identified (p = 0.022) indicates that financial factors substantially influence the accessibility and use of BRZ therapy among severe asthma patients. Treatments by biologics remains uncommon among patients with severe asthma^9,10^. A comparative examination of insurance types revealed that higher biologic treatment visits among privately insured individuals^11^. Furthermore, Cardet et al. reported that lower income independently contributed to adverse asthma outcomes, transcending factors such as education, perceived stress, race, and medication adherence^12^. The efficacy of biologics, specifically those targeting type 2 cytokines, has been well-established^13^. However, the observed disparities in access based on income and insurance type may hinder the broader application of these efficacious treatments, leading to suboptimal patient outcomes. While Japan’s public healthcare system provides uniform social security benefits to all citizens, the high cost of BRZ means that patients still incur some out-of-pocket expenses.

To overcome this economic barrier, some financial support programs may be needed to help patients with severe asthma access BRZ treatment, because appropriate use of biologics reduce exacerbation of asthma and cost for medication related to exacerbation^14,15^. For instance, in the United States, there are patient assistance programs that provide biologics to eligible patients with limited income or no insurance coverage. These programs may reduce the financial burden and improve adherence and satisfaction among patients who receive BRZ treatment. Similar programs may be beneficial for Japanese patients as well.

An alternative strategy to reduce the cost of biologics could be to adjust the treatment duration or reduce the dose for specific patients. Similar approaches have been successfully implemented in the treatment of rheumatic diseases. Studies have shown that spacing biologic treatments may be feasible for some patients, resulting in reduced costs and number of injections without compromising disease control^16^. Recent findings also support the safety and effectiveness of progressively reducing biologic drugs in rheumatoid arthritis patients in persistent remission^17^. Such strategies may be worth exploring in asthma treatment, with careful consideration of individual patient needs.

Some reports indicated that patients with access to a specialist are more likely to adhere to biologic treatment^18–20^. Race-ethnic differences are also identified as factors that influence the treatment and outcome of asthma^21,22^. Patient-related barriers to treatment adherence include a myriad of factors, such as understanding the need for treatment, confidence in clinicians or medication, and the severity of asthma. These barriers, while not directly investigated in our study, are important for personalized care approaches in the management of asthma.

There are some limitations in this study that need to be acknowledged. First, we used the SCR of BRZ as a proxy measure of its prescription rate, which may not reflect the actual number of patients who receive BRZ treatment in each prefecture. Second, we did not have access to individual patient data, such as age, sex, asthma severity, eosinophil count, comorbidities, and previous treatments, which may affect the decision to prescribe BRZ. Third, we did not consider other factors that may influence the prescription of BRZ, such as patient preference, physician preference, availability of biologics, and regional guidelines. Importantly, the national data utilized in this study does not provide information on the prescribing behaviors of individual physicians or referral patterns among physicians. This limitation suggests that the variations observed could be partly due to the differing tendencies of physicians to prescribe biologics, influenced by their specialization in severe asthma. Therefore, further studies with more detailed data are needed to confirm our findings and explore other factors related to the prescription of BRZ.

In conclusion, the interaction between income, insurance, and the access to and utilization of biologics for severe asthma treatment presents multifaceted challenges. Understanding these dynamics is crucial for healthcare providers, policymakers, and stakeholders to implement strategies that promote equitable access to these promising therapies. The evidence assembled in this study, alongside previous research, forms a compelling basis for further inquiry and policy action to mitigate these disparities.

## Methods

### National health insurance system and NDB open data

In Japan, the healthcare system is underpinned by public health insurance, where the patient’s financial contribution towards medical expenses is contingent upon their income level^23^. The residual costs are invoiced by individual medical institutions to the respective Claims Review and Reimbursement Organizations situated within each of Japan’s 47 prefectures, with payments being issued upon verification of claim validity. Furthermore, the fees for medical consultations, diagnostic procedures, and treatments are uniformly regulated across Japan, obligating all medical facilities to offer healthcare services at these established rates^24^.

The NDB was set up in 2009 under the “Act on Assurance of Medical Care for the Elderly People”. It’s among the world’s largest databases, gathering data on medical receipts since 2009 and on targeted health checkups and guidance since 2008. To encourage data use, Japan’s medical care state and health checkup outcomes were first shared as NDB open data in 2016. This open data includes seven key sections: “Medical Practice”, “Dental Practice”, “Dental Injuries and Diseases”, “Drugs”, “Specific Health Care Materials”, “Specific Health Examination (Laboratory Test Values)”, and “Specific Health Examination”. The data, available freely, is organized by fiscal year. As of 2022, it includes information up to the 2019 fiscal year.

### Study design

This research utilized an ecological study approach at the prefectural level, employing the same methodologies as those used in our prior study^25^. Using data on the number of prescriptions for oral medications generated from almost all insurance data, we examined differences in the propensity to prescribe BRZ between prefectures. We also examined the impact of differences in medical resources (number of specialists and hospitals) and conditions at diagnosis in each prefecture on prescribing trends.

### Data sources

SCRs for BRZ and omalizumab mepolizumab in 2020 were obtained from the Cabinet Office webpage “Regional Differences in Healthcare Delivery Status^26^. Information on average monthly earnings in each prefecture was obtained from the Basic Survey on Wage Structure published by the Ministry of Health, Labor and Welfare^27^. The obesity and smoking rates for those aged 40 and over in each prefecture were obtained from the 7th NDB^28^, the university enrollment rate in each prefecture was obtained from the Basic School Survey^29^, and the number of respiratory specialists and allergy specialists were obtained from the Japanese Respiratory Society, and Japanese Society of Allergology website.

### Indicators

As an indicator of the number of BRZ prescriptions, the standardized claim ratio (SCR) was calculated using the following formula^14^.

$$SCR= \frac{actual\, number \,of\, prescriptions}{expected \,number\, of \,prescriptions} \times 100,$$

$$Expected \,number \,of \,prescriptions= \sum \frac{A \times B}{C},$$

where $$A= number\, of\, residents\, in \,each\, prefecture \,by \,age\, and\, sex,$$ $$B= number \,of \,prescriptions\, in\, Japan\, by\, age \,and \,sex,$$ $$C= number\, of\, residents\, by\, age\, and \,sex\, in \,Japan.$$

The SCR is used to adjust for differences in the age and sex composition of each prefecture, and a score of 100 or more indicates that the number of cases is higher than the national average.

We also investigated the correlation between the SCR of BRZ and the SCRs of Omalizumab and Mepolizumab, as well as the average monthly income, smoking rate among those aged 40 and above, obesity rate among those aged 40 and above (BMI ≥ 25), the number of respiratory specialists certified by the Japanese Respiratory Society per population, the number of allergy specialists certified by the Japanese Society of Allergology per population, university enrollment rate, and the proportion of steroid-requiring exacerbations in each prefecture.

### Visualization of benralizumab standardized claim ratio (SCR) by prefecture

To visualize the regional distribution of benralizumab standardized claim ratios (SCR) across Japanese prefectures, we utilized Python along with the ‘japanmap’ and ‘matplotlib’ libraries. The dataset containing benralizumab SCR values for each prefecture was processed to ensure consistency and accuracy. A colormap was defined using ‘matplotlib’ to visually differentiate SCR values, and normalization techniques were applied to scale the SCR values appropriately. Using the ‘japanmap’ library, we generated a map of Japan where each prefecture was colored according to its SCR value. The intensity of the color represented the magnitude of the SCR, with a gradient from lighter to darker shades indicating lower to higher SCRs, respectively.

### Statistical evaluation

For statistical evaluation, JMP Pro 16 software (SAS Institute Inc.) was used for statistical analysis. The differences in the prescription patterns were assessed through descriptive statistics, which profiled the attributes of each prefecture. Furthermore, we utilized multiple regression analysis to explore the connections between the SCR and variables of clinical significance. Pearson’s correlation coefficient (*r*) and regression coefficient (*b*) were also calculated as supplementary analyses. No correlation was determined when $\left|r\right|$ < 0.2, weak correlation when 0.2 ≤ $\left|r\right|$  < 0.4, correlation when 0.4 ≤ $\left|r\right|$  < 0.7, and strong correlation when 0.7 ≤ $\left|r\right|$. The threshold for statistical significance was set at the 5% level.

### Ethical considerations

This study analyzed anonymized medical claims data from the NDB in Japan, which is provided by the Ministry of Health, Labour and Welfare. As the NDB data has been de-identified and made publicly available, this study was exempt from requiring informed consent or institutional review board approval per ethical guidelines.

## Data Availability

The data used in this study was obtained from the following websites: NDB open data: https://www.mhlw.go.jp/stf/seisakunitsuite/bunya/0000177182.html. National Cancer Registry: https://www.e-stat.go.jp/stat-search/files?page=1&toukei=00450173&tstat=000001133323b. The Japanese Respiratory Society: https://www.jrs.or.jp/search/specialist/index.php. Japanese Society of Allergology: https://www.jsaweb.jp/modules/ninteilist_general/.
